# Acetylation of aldehyde dehydrogenase ALDH1L2 regulates cellular redox balance and the chemosensitivity of colorectal cancer to 5-fluorouracil

**DOI:** 10.1016/j.jbc.2023.105090

**Published:** 2023-07-26

**Authors:** Chaoqun Li, Peng Teng, Shengbai Sun, Kaisa Cui, Surui Yao, Bojian Fei, Feng Ling, Zhaohui Huang

**Affiliations:** 1 Wuxi Cancer Institute, Affiliated Hospital of Jiangnan University, Wuxi, Jiangsu, China; 2 Laboratory of Cancer Epigenetics, Wuxi School of Medicine, Jiangnan University, Wuxi, Jiangsu, China; 3 Department of General Surgery, Affiliated Hospital of Jiangnan University, Wuxi, Jiangsu, China; 4 Chemical Genetics Laboratory, RIKEN Advanced Science Institute, Wako, Saitama, Japan

**Keywords:** acetylation, one carbon metabolism, ALDH1L2, SIRT3, drug resistance, posttranslational modification

## Abstract

Folate-mediated one-carbon metabolism (FOCM) is crucial in sustaining rapid proliferation and survival of cancer cells. The folate cycle depends on a series of key cellular enzymes, including aldehyde dehydrogenase 1 family member L2 (ALDH1L2) that is usually overexpressed in cancer cells, but the regulatory mechanism of ALDH1L2 remains undefined. In this study, we observed the significant overexpression of ALDH1L2 in colorectal cancer (CRC) tissues, which is associated with poor prognosis. Mechanistically, we identified that the acetylation of ALDH1L2 at the K70 site is an important regulatory mechanism inhibiting the enzymatic activity of ALDH1L2 and disturbing cellular redox balance. Moreover, we revealed that sirtuins 3 (SIRT3) directly binds and deacetylates ALDH1L2 to increase its activity. Interestingly, the chemotherapeutic agent 5-fluorouracil (5-Fu) inhibits the expression of SIRT3 and increases the acetylation levels of ALDH1L2 in colorectal cancer cells. 5-Fu-induced ALDH1L2 acetylation sufficiently inhibits its enzymatic activity and the production of NADPH and GSH, thereby leading to oxidative stress-induced apoptosis and suppressing tumor growth in mice. Furthermore, the K70Q mutant of ALDH1L2 sensitizes cancer cells to 5-Fu both *in vitro* and *in vivo* through perturbing cellular redox and serine metabolism. Our findings reveal an unknown 5-Fu-SIRT3-ALDH1L2 axis regulating redox homeostasis, and suggest that targeting ALDH1L2 is a promising therapeutic strategy to sensitize tumor cells to chemotherapeutic agents.

Folate-mediated one-carbon metabolism (FOCM) is indispensable for cellular homeostasis because it plays a key role in nucleotide synthesis, methylation, and reductive metabolism, supporting inappropriate cell proliferation (1, 2). Therefore, anti-folate drugs targeting one-carbon metabolism have long been used in cancer treatment, and some key one-carbon metabolism enzymes have been considered promising therapeutic targets for human cancers (3, 4, 5).

Aldehyde dehydrogenase 1 family member L1 (ALDH1L1) and ALDH1L2, two key folate-metabolizing enzymes, control the overall flux of one-carbon group and cell proliferation in folate-dependent biosynthetic pathways, through converting 10-formyltetrahydrofolate to tetrahydrofolate and CO_2_, simultaneously producing NADPH (6). ALDH1L2 was relatively recently discovered and little was known about its role in physiological and pathological conditions. In contrast to its cytosolic homolog ALDH1L1, ALDH1L2 is usually overexpressed in cancer cells, suggesting its potential role in tumorigenesis and progression. It has been reported that ALDH1L2 catalyzed reaction is an important source of NADPH in mitochondria (7), which links this enzyme with cancer metastasis (8). However, the biological significance of this enzyme and how it affects colorectal cancer (CRC) tumorigenesis and progression are not completely clear. Also, the regulatory mechanism of ALDH1L2 in cancer is also largely unknown.

Posttranslational modifications (PTMs) of proteins remarkably expand proteome diversity and play critical roles in mammalian cells by regulating gene expression, protein stability, and enzyme activities. The activity of metabolic enzymes is modulated by various types of PTMs, which allow cancer cells to respond quickly to external environmental signals and stresses, promoting cancer progression. Protein lysine acetylation is a conserved PTM, reversibly catalyzed by acetyltransferases and deacetylases (9). Proteomic studies of mouse livers have shown that more than 20% of mitochondrial proteins are acetylated, including many metabolic enzymes (10). Some in-depth studies have been carried out to investigate the role of acetylation in these metabolic enzymes (11, 12). However, the effects of PTMs on the functions of ALDH1L2 remain to be elucidated.

In this study, we demonstrated that ALDH1L2 is overexpressed in CRC, and the acetylation modification of ALDH1L2 at K70 site suppresses its activity. Sirtuins 3 (SIRT3), an NAD^+^-dependent deacetylase, could increase ALDH1L2 activity by deacetylating it. Additionally, the chemotherapeutic agent 5-fluorouracil (5-Fu) inhibits SIRT3 expression and promotes ALDH1L2 acetylation, which decreases ALDH1L2 activity and NADPH/NADP^+^ ratio. Furthermore, we indicated that the K70Q mutant of ALDH1L2 disturbs cellular redox and serine metabolism, inhibiting CRC cell proliferation and sensitizing CRC cells to 5-Fu treatment both *in vitro* and *in vivo*.

## Results

### ALDH1L2 is upregulated in CRC and promotes CRC proliferation

**Figure 1:**
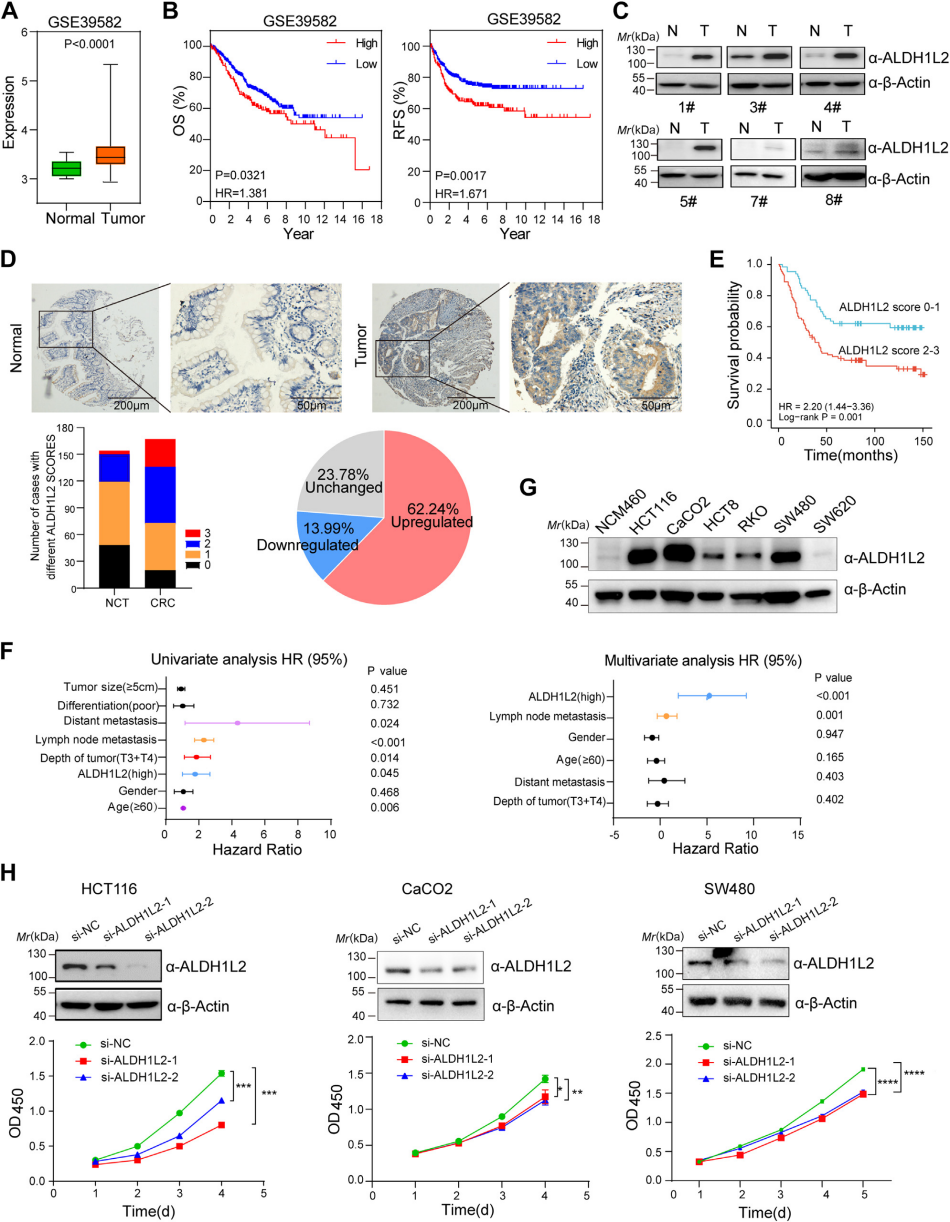
**ALDH1L2 is upregulated in CRC and promotes CRC proliferation.***A*, relative expression levels of ALDH1L2 in CRC dataset of GSE39582. *B*, the effects of ALDH1L2 expression on the overall survival (OS) (*left*) and recurrence free survival (RFS) (*right*) of patients with CRC in GSE39582 database. The log-rank test was used to calculate *p*-values. *C*, six representative Western blotting analyses of ALDH1L2 protein expression in six represented peritumoral/tumor tissue pairs from CRC patients. These images were extracted from Fig. S1*A*. *D*, ALDH1L2 protein expression levels were detected and analyzed in CRC using IHC staining. IHC analyses were performed in 167 human CRC cases, including 143 paired tumor tissues and adjacent normal tissues. *E*, patients with high ALDH1L2 expression (score 2–3) had poorer OS than those with low ALDH1L2 expression (score 0–1). The log-rank test was used to calculate *p*-values. *F*, Cox univariate and multivariate regression analyses in CRC patients. The hazard ratio (HR) and 95% confidence interval (CI) are plotted for each factor. *G*, ALDH1L2 protein expressions in a panel of CRC cell lines and a normal colon cell line NCM460. *H*, the effects of ALDH1L2 knockdown on the cell proliferation of HCT116, CaCO2, and SW480 cells. The knockdown efficiency of ALDH1L2 by siRNA in cells was verified by Western blotting. Data are shown as mean ± S.D (n = 3). ∗*p*< 0.05; ∗∗*p* < 0.01; ∗∗∗*p*< 0.001. ALDH1L2, aldehyde dehydrogenase 1 family member; CRC, colorectal cancer; IHC, immunohistochemistry.

**Figure S1:**
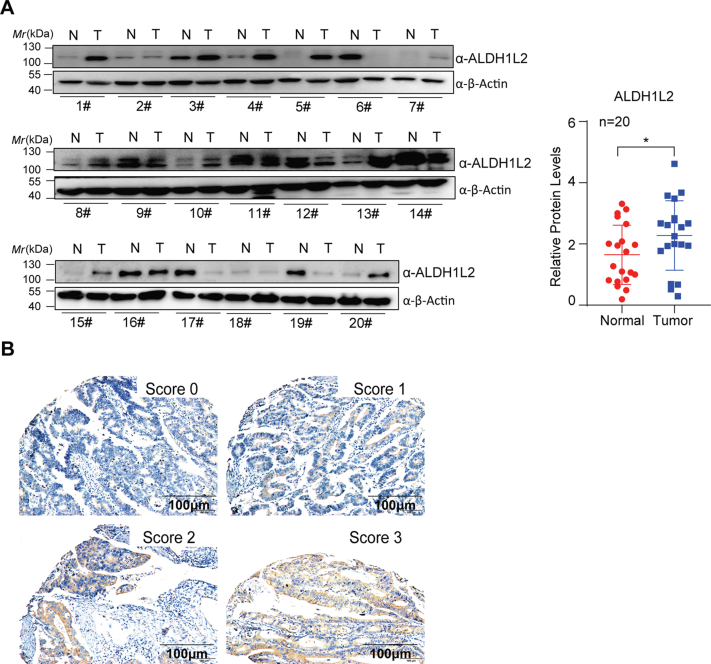
*A*, Western blotting analysis of ALDH1L2 expression in 20 pairs of colorectal cancer tissues and matched paracancerous tissue surgically obtained from the same patients (*left*). Quantitative analysis of ALDH1L2 expression was shown in tumor tissues compared to the matching peritumoral tissues (*right*). *B*, scores indicated ALDH1L2 levels in representative tumor tissues as determined by IHC staining. The scores were calculated as the staining intensity and the percentage of stained cells. Scale bars as marked. ∗*p* < 0.05.

To explore the role of ALDH1L2 in CRC progression, we first demonstrated that *ALDH1L2* is overexpressed in CRC tissues and correlated with poor prognosis by analyzing the mRNA expression of *ALDH1L2* based on the Gene Expression Omnibus database (Fig. 1, *A* and *B*). We then examined ALDH1L2 protein expression levels in 20 paired CRC tissues using Western blotting. Of these tumors, 11 showed higher ALDH1L2 expression compared with adjacent noncancerous tissues (NCTs) (Figs. 1*C* and S1*A*). Through immunohistochemistry (IHC) staining of 143 paired CRC and NCT samples, we also found that more than half of CRCs (62.2%, 89/143) displayed elevated ALDH1L2 expression compared with their matched NCTs (Figs. 1*D* and S1*B*), and patients with higher ALDH1L2 expression showed poorer overall survival (Fig. 1*E*). Besides, univariate and multivariate Cox proportional hazards analyses demonstrated that ALDH1L2 was an independent prognostic factor for CRC patients (Fig. 1*F*).

We also observed increased ALDH1L2 protein expression in a panel of CRC cell lines compared with NCM460, a normal colon cell line (Fig. 1*G*). In order to assess the potential functional role of ALDH1L2 in CRC, we depleted ALDH1L2 using siRNA in HCT116, CaCO2, and SW480 cell lines with relatively high ALDH1L2 expression (Fig. 1*H*). Cell counting kit-8 assays demonstrated that knockdown of ALDH1L2 inhibited CRC proliferation (Fig. 1*H*). Thus, these data suggest that high ALDH1L2 expression is essential for CRC cell proliferation.

### ALDH1L2 is acetylated at K70

**Figure 2:**
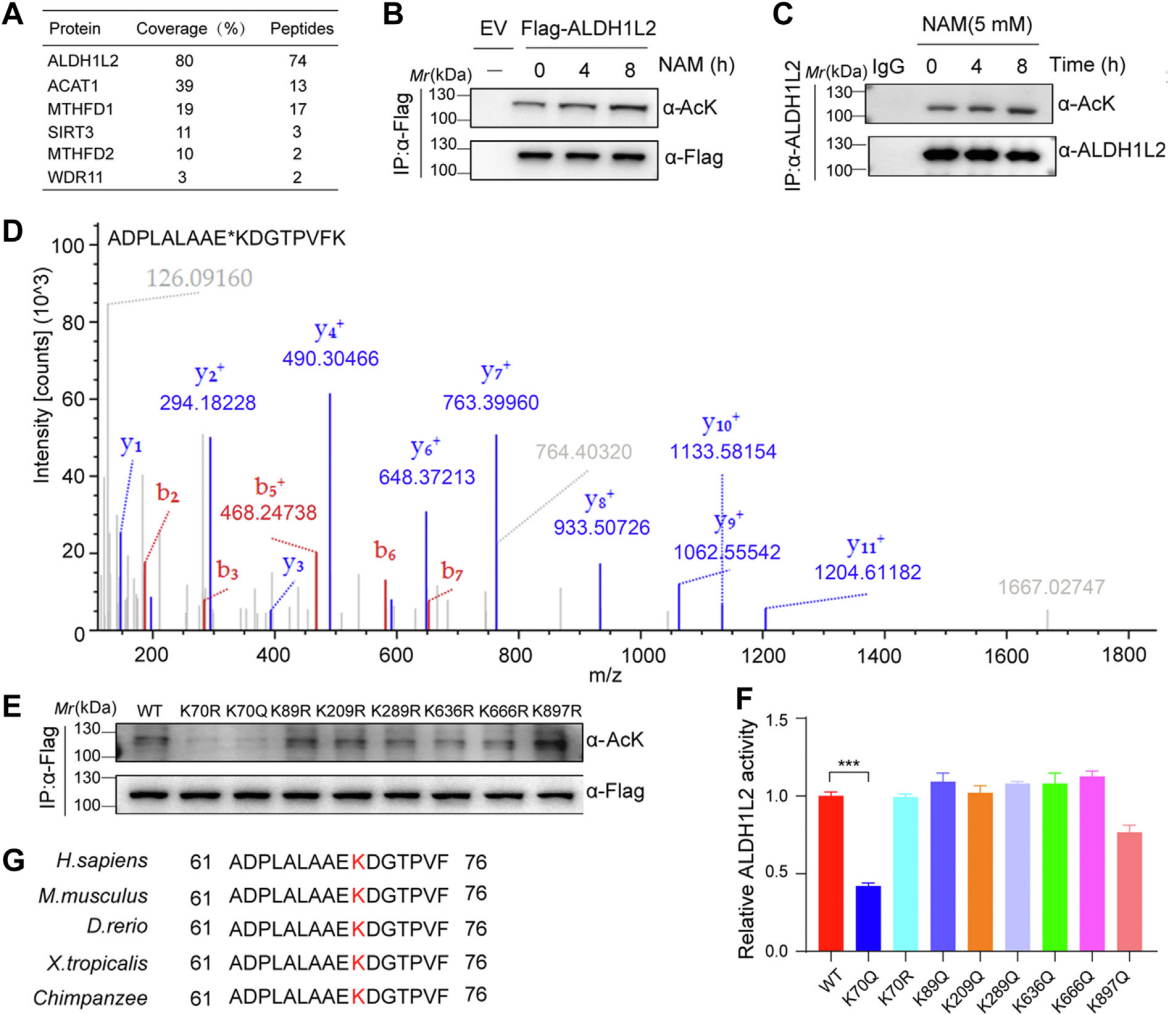
**K70 acetylation of ALDH1L2 inhibits the enzyme activity of ALDH1L2.***A*, searching for potential ALDH1L2-interacting proteins by mass spectrometry. The ALDH1L2-associated proteins were immunoprecipitated from HEK293T cells transfected with Flag-ALDH1L2 using anti-Flag M2 beads and were analyzed by mass spectrometry. *B*, acetylation of ectopically expressed ALDH1L2 proteins was analyzed in HEK293T cells treated with 5 mM NAM. Flag-ALDH1L2 was transfected and immunoprecipitated from HEK293T cells, and ALDH1L2 acetylation was examined with a pan-acetylated lysine (anti-AcK) antibody. *C*, the endogenous ALDH1L2 acetylation was analyzed in HCT116 cells treated with NAM. ALDH1L2 was immunoprecipitated and its acetylation was examined with anti-AcK antibody. *D*, identification of ALDH1L2 acetylated peptides around K70 site by mass spectrometry. *E* and *F*, mapping the major acetylated sites of ALDH1L2. ALDH1L2 WT and seven K-R mutants were transfected into HEK293T cells. Flag-ALDH1L2 proteins were purified by IP using anti-Flag M2 beads. Acetylation levels of purified ALDH1L2 proteins were detected using Western blotting (*E*). Enzymatic activity of purified ALDH1L2 proteins was determined by a spectrophotometrical enzymatic assay (*F*). Error bars indicate the means ± SD (n = 3); ∗∗∗*p* < 0.001. *G*, conserved sequences analyses of ALDH1L2 in different species. The sequences of ALDH1L2 around K70 site from different species were aligned. ALDH1L2, aldehyde dehydrogenase 1 family member; IP, immunoprecipitation; NAM, nicotinamide.

ALDH1L2 is defined as a metabolic enzyme located in the mitochondria. Aiming to explore its exact role in cellular metabolism, we transfected HEK293T cells with Flag-tagged ALDH1L2, and then screened potential ALDH1L2 interacting proteins using immunoprecipitation (IP) and mass spectrometry analyses. Notably, we identified SIRT3, an NAD+-dependent deacetylase, as an ALDH1L2-associated protein (Fig. 2*A*). Since most of the mitochondrial proteins are reported to be acetylated and more than 90% of SIRT3 interactomes are acetylated proteins (13, 14), we speculated the existence of ALDH1L2 acetylation. Acetylation, a key PTM, plays a significant role in regulating the structure and function of metabolic enzymes. To determine whether ALDH1L2 is modified by acetylation, we overexpressed Flag-ALDH1L2 in HEK293T cells following treatment with nicotinamide (NAM), a pan-sirtuin family inhibitor, to determine whether ALDH1L2 is modified by acetylation. The results showed that ALDH1L2 was indeed acetylated and its acetylation levels were enhanced by NAM treatment in a time-dependent manner (Fig. 2*B*). Also, ALDH1L2 acetylation could be detected with IP of endogenous ALDH1L2 in HCT116 cells (Fig. 2*C*).

**Figure S2:**
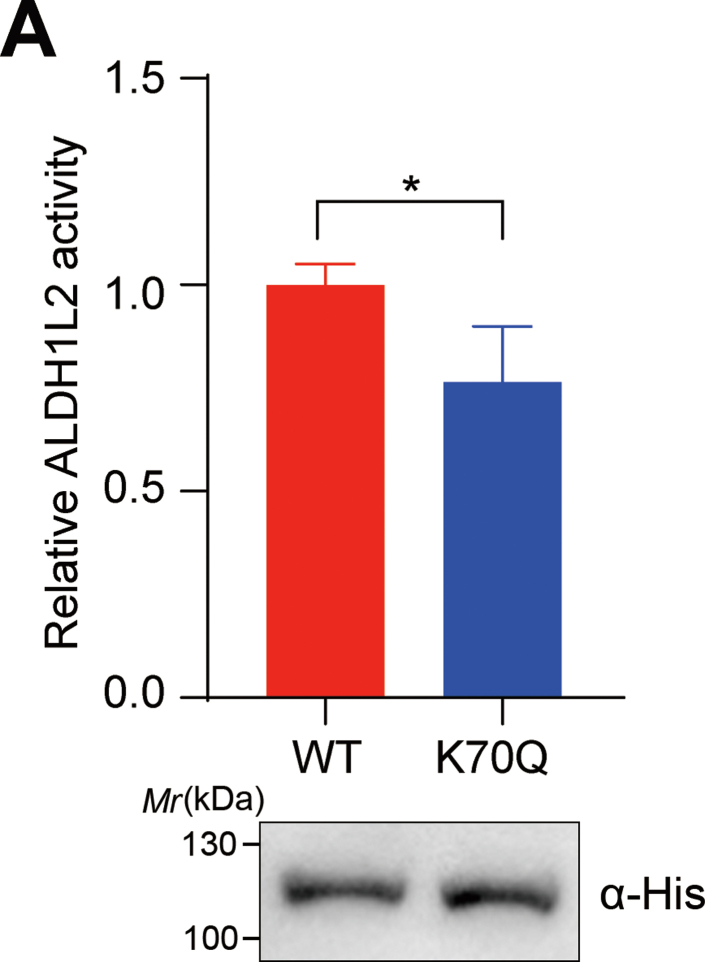
*A*, enzymatic activities of recombinant His-ALDH1L2 WT and K70Q mutant proteins were determined by a spectrophotometrical enzymatic assay (*upper)*. An anti-His western blotting shows that equivalent ALDH1L2 protein levels were used (*down)*. Error bars indicate the means ± SD (n = 3); ∗*p* < 0.05.

To further identify the main acetylation sites on ALDH1L2, we purified acetylated ALDH1L2 protein from HEK293T cells and examined its acetylation sites by mass spectrometry. Seven lysine residues (K70, K89, K209, K289, K636, K666, and K897) were identified as potential acetylated sites (Fig. 2*D*) and were then mutated to arginine (R, to mimic deacetylated state) and glutamine (Q, to mimic acetylated state). These ALDH1L2 mutants were overexpressed in HEK293T cells and immunoprecipitated to evaluate their effects on the acetylation and activity ALDH1L2. Of these mutants, K70R mutant displayed a remarkable decrease in overall acetylation levels and K70Q showed lower enzymatic activity than WT (Fig. 2, *E* and *F*). To further demonstrate the effect of K70 acetylation on ALDH1L2 activity, we expressed and purified recombinant WT and K70Q ALDH1L2. Enzyme activity assays showed that K70Q mutation decreases ALDH1L2 activity (Fig. S2*A*). Moreover, K70 is a highly conserved site on ALDH1L2 across several mammalian species (Fig. 2*G*). The above data demonstrate that ALDH1L2 could be acetylated and the acetylation at K70 abolishes ALDH1L2 enzymatic activity.

### SIRT3 deacetylates ALDH1L2

**Figure 3:**
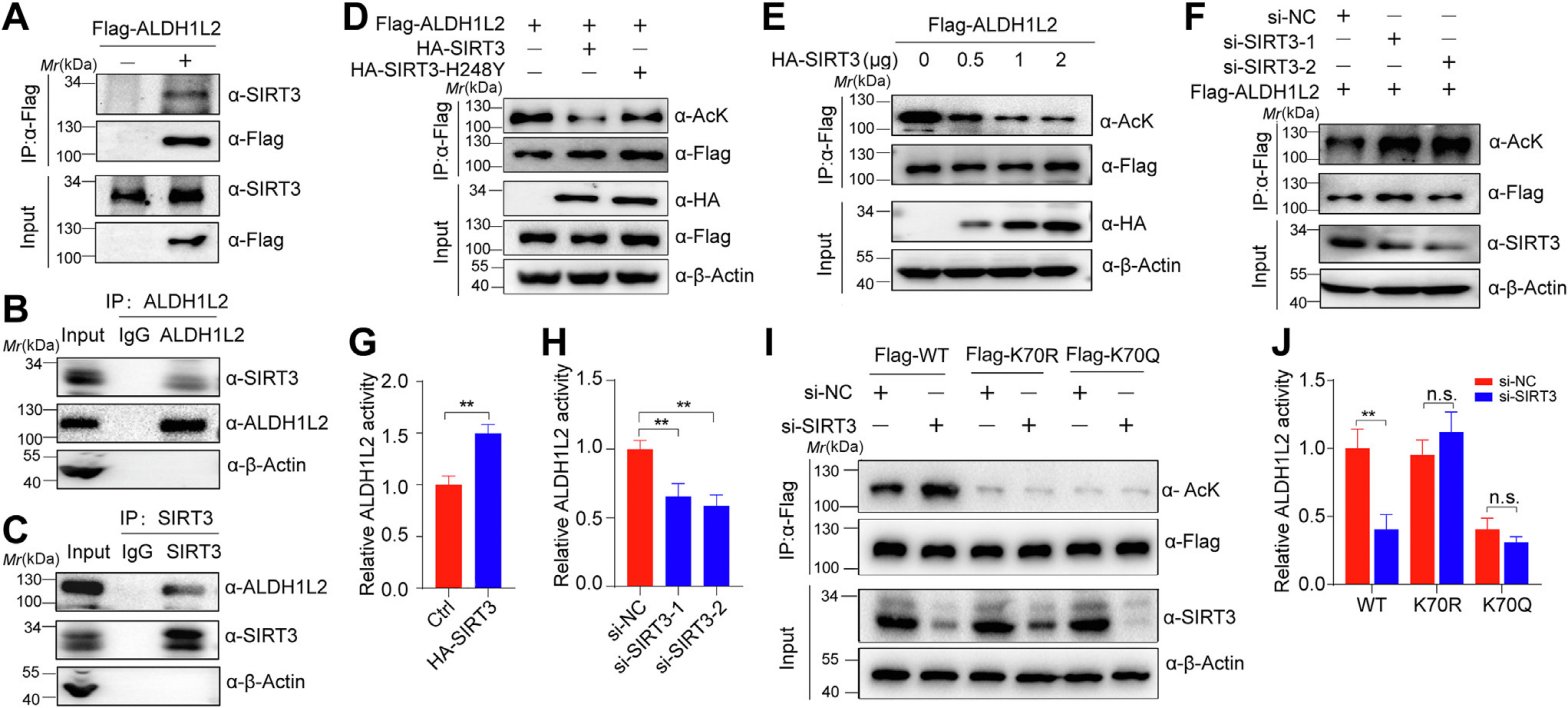
**SIRT3 deacetylates ALDH1L2**. *A*, exogenous interaction between ALDH1L2 and SIRT3. Flag-ALDH1L2 was transfected into HEK293T cells and immunoprecipitates with anti-Flag M2 beads were detected with anti-SIRT3 antibody. *B* and *C*, endogenous interaction between ALDH1L2 and SIRT3. HCT116 cell lysates were immunoprecipitated with control IgG, anti-SIRT3, or anti-ALDH1L2, and the precipitated proteins were detected with anti-ALDH1L2 or anti-SIRT3, respectively. *D*, effects of SIRT3 or its dead mutant on the acetylation of ALDH1L2. HEK293T cells were cotransfected with Flag-ALDH1L2, HA-SIRT3, or HA-SIRT3-H248Y (H248Y), followed by deacetylation assays. *E*, effects of SIRT3 overexpression on the acetylation level of ALDH1L2. HEK293T cells were cotransfected with Flag-ALDH1L2 alone or with different amounts of HA-SIRT3 plasmid, followed by deacetylation assays. *F*, effects of SIRT3 knockdown on ALDH1L2 acetylation. Flag-ALDH1L2 was cotransfected with si-SIRT3 into HEK293T cells. Immunoprecipitated ALDH1L2 was analyzed with anti-AcK antibody. *G*, effects of SIRT3 overexpression on ALDH1L2 activity. HEK293T cells were transfected with Flag-ALDH1L2 and/or HA-SIRT3. Immunoprecipitated ALDH1L2 was subjected to enzymatic activity analyses. *H*, effects of SIRT3 knockdown on ALDH1L2 activity. Flag-ALDH1L2 was cotransfected with si-SIRT3 into HEK293T cells. Immunoprecipitated ALDH1L2 activity was detected by enzymatic activity analyses. *I*, effects of SIRT3 knockdown on the acetylation levels of ALDH1L2 WT and K70R/K70Q mutants. Flag-WT ALDH1L2 or its mutants (K70R and K70Q) were cotransfected with si-SIRT3 into HEK293T cells. Flag-ALDH1L2 was then immunoprecipitated and its acetylation was examined. *J*, the activities of ALDH1L2 WT, K70R and K70Q mutants in HEK293T cells after knockdown of SIRT3. Flag-WT ALDH1L2 or its mutants (K70R and K70Q) were cotransfected with si-SIRT3 into HEK293T cells. The activity of immunoprecipitated Flag-WT ALDH1L2 or its mutants was detected by a spectrophotometrical enzymatic assay. Data are shown as mean ± S.D (n = 3). ∗∗*p* < 0.01, n.s., not significant. ALDH1L2, aldehyde dehydrogenase 1 family member L2; IgG, immunoglobulin; SIRT3, sirtuins 3.

**Figure S3:**
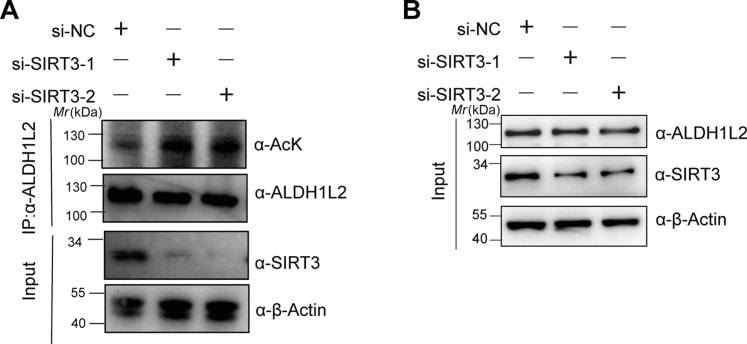
*A*, effects of SIRT3 knockdown on endogenous ALDH1L2 acetylation. HCT116 cells were transfected with si-SIRT3. Immunoprecipitated ALDH1L2 was analyzed with anti-AcK antibodies. *B*, effects of SIRT3 knockdown on ALDH1L2 protein level. HCT116 cells were transfected with si-SIRT3 and cell lysates were detected with indicated antibodies.

NAM treatment increased ALDH1L2 acetylation, implying that NAD+-dependent sirtuins are involved in ALDH1L2 deacetylation. Besides, we have identified ALDH1L2 as a SIRT3-interacting protein (Fig. 2*A*). These results lead us to examine whether SIRT3 interacts with and deacetylates ALDH1L2. We first conducted co-immunoprecipitation (Co-IP) experiments to test this hypothesis and found that Flag-ALDH1L2 could pull down SIRT3 (Fig. 3*A*). We also proved the interaction between SIRT3 and ALDH1L2 at the endogenous level (Fig. 3, *B* and *C*). Furthermore, we confirmed that overexpression of SIRT3, other than SIRT3 enzymatically defective mutant SIRT3-H248Y, dramatically reduced ALDH1L2 acetylation levels (Fig. 3*D*), and SIRT3 deacetylated ALDH1L2 in cells in a dose-dependent manner (Fig. 3*E*). Conversely, knocking down SIRT3 using siRNA led to increased ALDH1L2 acetylation levels (Figs. 3*F* and S3*A*). Since acetylation inhibits ALDH1L2 activity, we explored whether SIRT3 regulates ALDH1L2 activity and observed that SIRT3 overexpression increased, whereas SIRT3 knockdown reduced ALDH1L2 activity (Fig. 3, *G* and *H*). However, no obvious effect of SIRT3 on the acetylation and activity of K70R and K70Q mutants of ALDH1L2 was observed (Fig. 3, *I* and *J*). Besides, SIRT3 manipulation did not alter ALDH1L2 protein levels (Fig. S3*B*). These data indicate that SIRT3-mediated ALDH1L2 acetylation regulates its enzymatic activity.

### 5-Fu treatment decreases SIRT3 expression and promotes apoptosis

**Figure 4:**
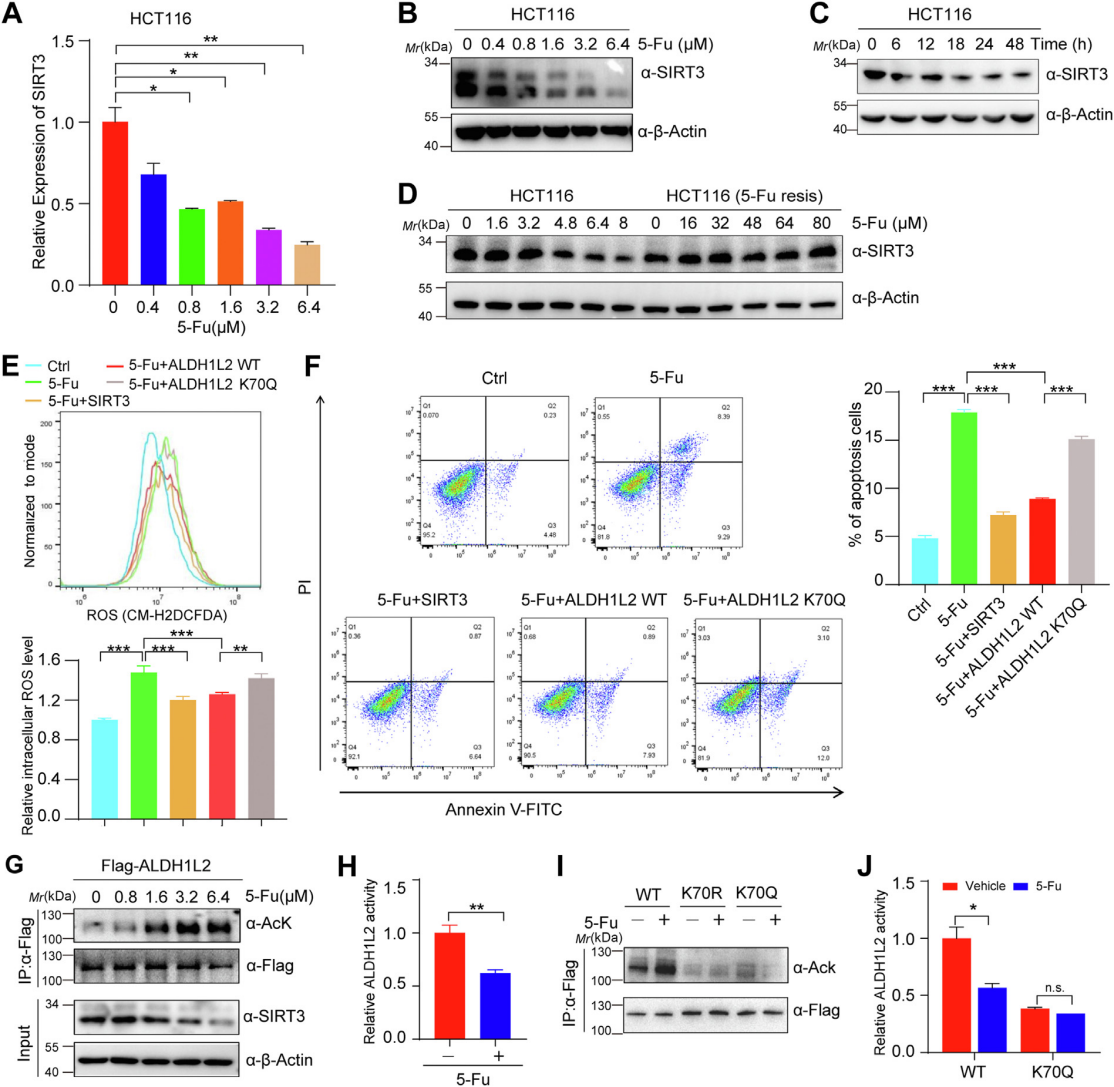
**5-Fu treatment decreases SIRT3 expression and promotes apoptosis.***A*, the effects of 5-Fu treatment on *SIRT3* mRNA levels in HCT116 cells. HCT116 cells were treated with different concentrations of 5-Fu for 48 h. *B* and *C*, the effects of 5-Fu treatment on SIRT3 protein levels in HCT116 cells. HCT116 cells were treated with 5-Fu for 48 h at different concentrations (*B*) or at a concentration of 0.8 μM for indicated duration (*C*). *D*, SIRT3 protein levels in HCT116 cells and 5-Fu-resistant HCT116 cells treated with different concentrations of 5-Fu. HCT116 cells and 5-Fu-resistant HCT116 cells were treated with 5-Fu for 48 h with indicated concentrations. *E*, ROS levels of 5-Fu-treated HCT116 cells with or without SIRT3 overexpression. ROS levels were determined by flow cytometry after DCFH-DA staining and quantified by the mean intensity of DCF fluorescence. Cells were treated by 5-Fu for 48 h. *F*, the effects of SIRT3 or ALDH1L2 overexpression on the 5-Fu-induced apoptosis in HCT116 cells. *G*, acetylation levels of ectopically expressed Flag-ALDH1L2 were detected in HEK293T cells treated with 5-Fu for 48 h at different concentrations. *H*, the effects of 5-Fu treatment on the enzymatic activity of ALDH1L2. HEK293T cells were transfected with Flag-ALDH1L2, followed by treatment with or without 5-Fu. Immunoprecipitated Flag-ALDH1L2 activity was detected by a spectrophotometrical enzymatic assay. *I*, the effects of 5-Fu on the acetylation levels of ALDH1L2 WT and K70R/K70Q mutants. Flag-WT ALDH1L2 or its mutants (K70R and K70Q) were transfected into HEK293T cells, followed by treatments with 5-Fu. Flag-ALDH1L2 was then immunoprecipitated and its acetylation was examined. *J*, the activities of ALDH1L2 WT and K70Q mutant in HEK293T cells treated with 5-Fu. The activity of immunoprecipitated Flag-WT ALDH1L2 or its K70Q mutant from HEK293T cells was detected by a spectrophotometrical enzymatic assay. Data are shown as mean ± S.D (n = 3). ∗*p* < 0.05; ∗∗*p* < 0.01, ∗∗∗*p* < 0.001, n.s., not significant. 5-Fu, 5-fluorouracil; ALDH1L2, aldehyde dehydrogenase 1 family member L2; DCFH-DA, dichloro-dihydro-fluorescein diacetate; ROS, reactive oxygen species; SIRT3, sirtuins 3.

**Figure S4:**
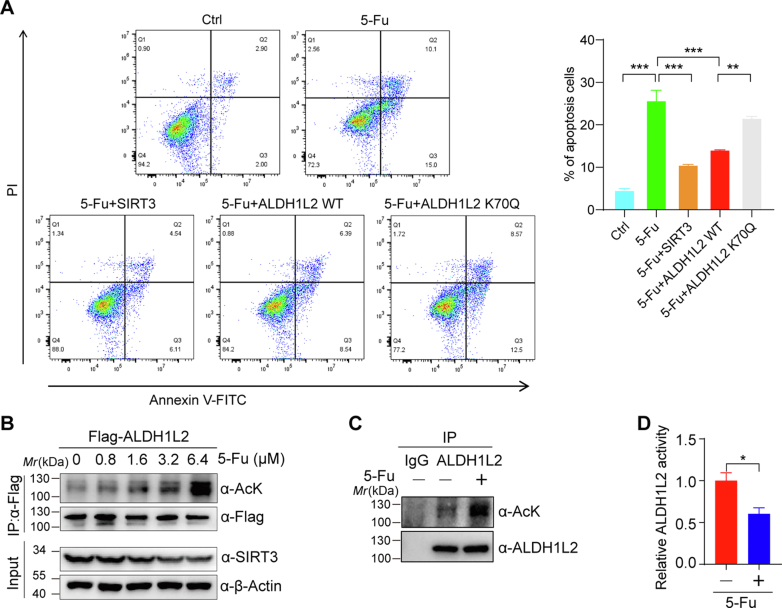
*A*, the effects of SIRT3 or ALDH1L2 overexpression on the 5-Fu-induced apoptosis in HCT8 cells. *B*, Acetylation levels of ectopically expressed Flag-ALDH1L2 were detected in HCT8 cells treated with 5-Fu for 48 h at different concentrations. *C*, Acetylation levels of endogenous ALDH1L2 were detected in HCT116 cells treated with 5-Fu for 48 h. ALDH1L2 was immunoprecipitated from HCT116 cells treated with or without 5-Fu and its acetylation was examined with anti-AcK antibodies. *D*, the effects of 5-Fu treatment on the enzymatic activity of endogenous ALDH1L2. HCT116 cells were lysed after treatment with or without 5-Fu. The activities of immunoprecipitated ALDH1L2 were detected by a spectrophotometrical enzymatic assay. Data are shown as mean ± S.D (n = 3). ∗*p* < 0.05; ∗∗*p* < 0.01, ∗∗∗*p* < 0.001.

In addition to the cytotoxic effect produced by inhibiting thymidylate synthesis and misincorporating its metabolites into RNA and DNA, 5-Fu could also induce oxidative stress and alter cell metabolism (15, 16). Considering that SIRT3 is one of the key regulators of cell metabolism and responds to various oxidative stress signals and injury signals, we next explored whether 5-Fu affects SIRT3 expression and thus cell metabolism. quantitative reverse transcription-PCR (qRT-PCR) results showed a significant decrease in *SIRT3* expression in HCT116 cells treated with 5-Fu (Fig. 4*A*). Moreover, in 5-Fu-treated HCT116 cells, the protein levels of SIRT3 were also reduced in a dose- or time-dependent manner (Fig. 4, *B* and *C*). Interestingly, no obvious change was observed in SIRT3 expression in 5-Fu-resistant HCT116 cells upon 5-Fu exposure, suggesting that SIRT3 was associated with the response of CRC cells to 5-Fu treatment (Fig. 4*D*). In addition, we also found that 5-Fu treatment induces oxidative stress and apoptosis, which can be rescued by ectopic expression of SIRT3 or ALDH1L2, not ALDH1L2 K70Q mutant (Figs. 4, *E* and *F* and S4*A*). These results suggest that SIRT3 is a new target for 5-Fu, and the cytotoxicity of 5-Fu may be partly mediated by inhibiting SIRT3-regulated cell metabolism.

Since ALDH1L2 is a new substrate of SIRT3, we measured the effects of 5-Fu on ALDH1L2. The results showed that 5-Fu treatment could increase ALDH1L2 acetylation (Figs. 4*G* and S4, *B* and *C*) and decrease its activity (Figs. 4*H* and S4*D*), but have no obvious effects on ALDH1L2 K70Q mutant (Fig. 4, *I* and *J*), indicating that 5-Fu promotes ALDH1L2 acetylation and inhibits its activity by downregulating SIRT3 expression, which may be involved in oxidative stress-induced cell apoptosis.

### ALDH1L2 K70Q mutant disturbs cellular redox balance and sensitizes cancer cells to 5-Fu

**Figure 5:**
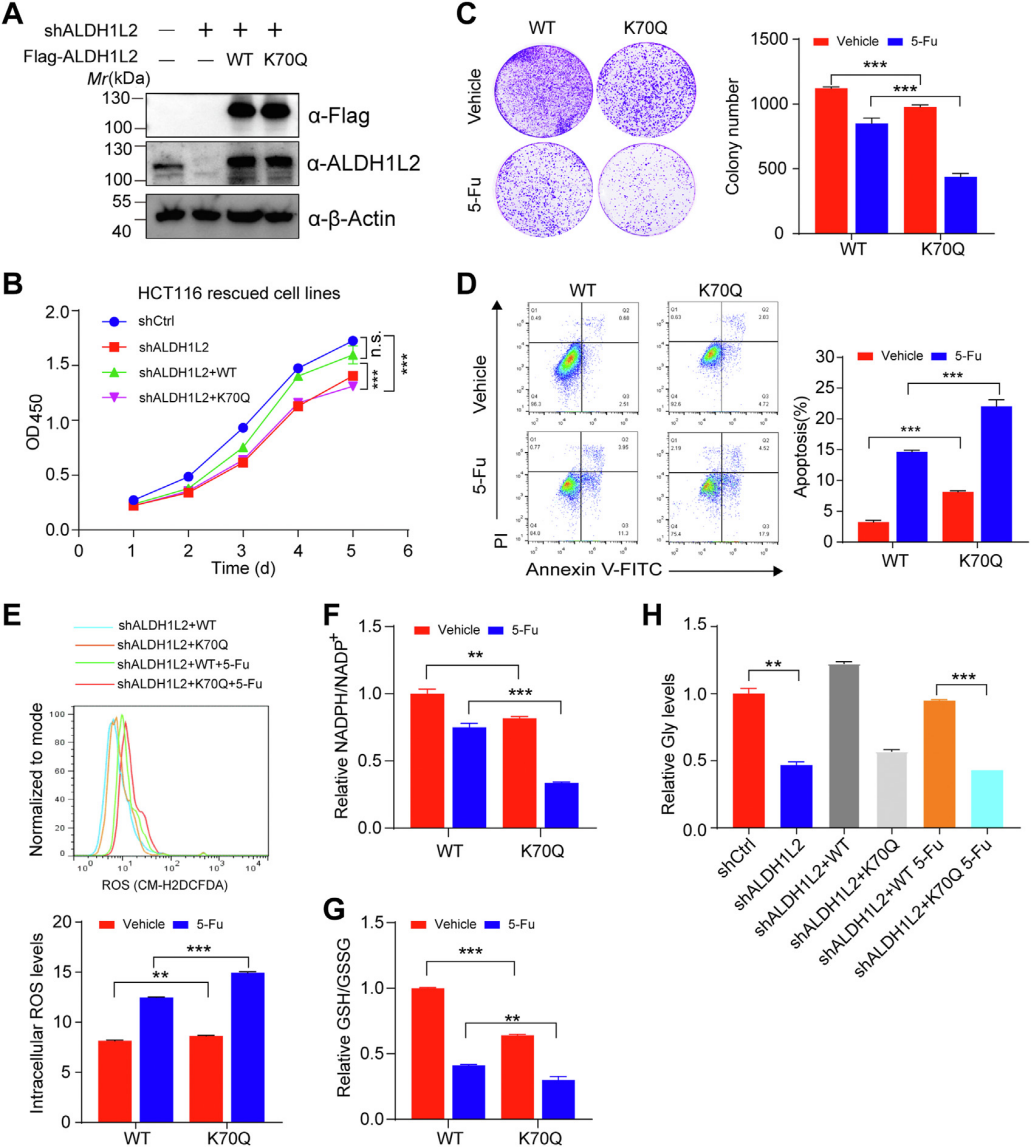
**ALDH1L2 K70Q mutant disturbs cellular redox balance and sensitizes cancer cells to 5-Fu.***A*, identification of reconstituted HCT116 cell lines. ALDH1L2-depleted HCT116 cells were reconstituted with empty vector, shRNA-resistant Flag-tagged WT and K70Q mutant of ALDH1L2. Total cell lysates were prepared and detected by Western blotting. *B*, four cell lines from (*A*) were subjected to CCK-8 assays. *C* and *D*, colony formation (*C*) and apoptosis (*D*) analyses in reconstituted ALDH1L2 WT or K70Q cells treated with 5-Fu. *E*–*G*, ROS levels (*E*), NADPH/NADP^+^ ratio (*F*) and GSH/GSSG ratio (*G*) in cells were measured in reconstituted ALDH1L2 WT or K70Q cells treated with 5-Fu. *H*, cellular glycine levels were measured in four cell lines from (*A*) by GC-MS. Data are shown as mean ± S.D (n = 3). ∗*p* < 0.05; ∗∗*p* < 0.01; ∗∗∗*p* < 0.001; n.s., not significant. 5-Fu, 5-fluorouracil; ALDH1L2, aldehyde dehydrogenase 1 family member L2; CCK-8, cell counting kit-8; ROS, reactive oxygen species.

To further characterize the role of K70 acetylation of ALDH1L2, we depleted the endogenous ALDH1L2 and reconstituted with shRNA-resistant Flag-tagged ALDH1L2 WT or K70Q in HCT116 cells (Fig. 5*A*). ALDH1L2 knockdown inhibited the proliferation of HCT116 cells, which can be rescued by reexpression of ALDH1L2 WT, but not K70Q mutant (Fig. 5*B*), indicating that K70 acetylation reduces the activity of ALDH1L2 and impairs its ability to sustain CRC cell proliferation. Interestingly, colony survival assays showed that ALDH1L2 K70Q cells were more sensitive to 5-Fu treatment than ALDH1L2 WT rescued cells (Fig. 5*C*); apoptosis assays also demonstrated that 5-Fu treatment induced stronger apoptosis in ALDH1L2 K70Q cells than in WT rescued cells (Fig. 5*D*). Given that ALDH1L2 is related to NADPH production and the resistance to oxidative damages, we detected reactive oxygen species (ROS) levels, NADPH/NADP^+^ and GSH/GSSG ratios in reconstituted HCT116 cells and found that ALDH1L2 WT rescued cells displayed higher NADPH/NADP^+^ and GSH/GSSG ratios than ALDH1L2 K70Q cells (Fig. 5, *F* and *G*). At the same time, ALDH1L2 K70Q cells also showed higher cellular ROS and apoptosis levels (Fig. 5, *D* and *E*). Considering the fact that 5-Fu promotes ALDH1L2 acetylation *via* downregulating SIRT3 expression, we examined the effects of ALDH1L2 acetylation on CRC cells in response to 5-Fu treatment. As expected, ALDH1L2 K70Q cells were more sensitive to 5-Fu in comparison with WT rescued cells, as demonstrated by the decreased NADPH/NADP^+^ and GSH/GSSG ratios, and elevated ROS and apoptosis levels (Fig. 5, *D–G*). In addition, we also observed that the glycine level was decreased in ALDH1L2 K70Q cells compared with ALDH1L2 WT cells (Fig. 5*H*), suggesting that acetylated ALDH1L2 may disturb the FOCM pathway and inhibit serine/glycine conversion. Taken together, these data demonstrate that K70Q mutant of ALDH1L2 suppresses CRC cell proliferation and sensitizes cancer cells to 5-Fu through disturbing cellular redox balance.

### ALDH1L2 K70Q mutant inhibits CRC growth and promotes 5-Fu sensitivity *in vivo*

**Figure 6:**
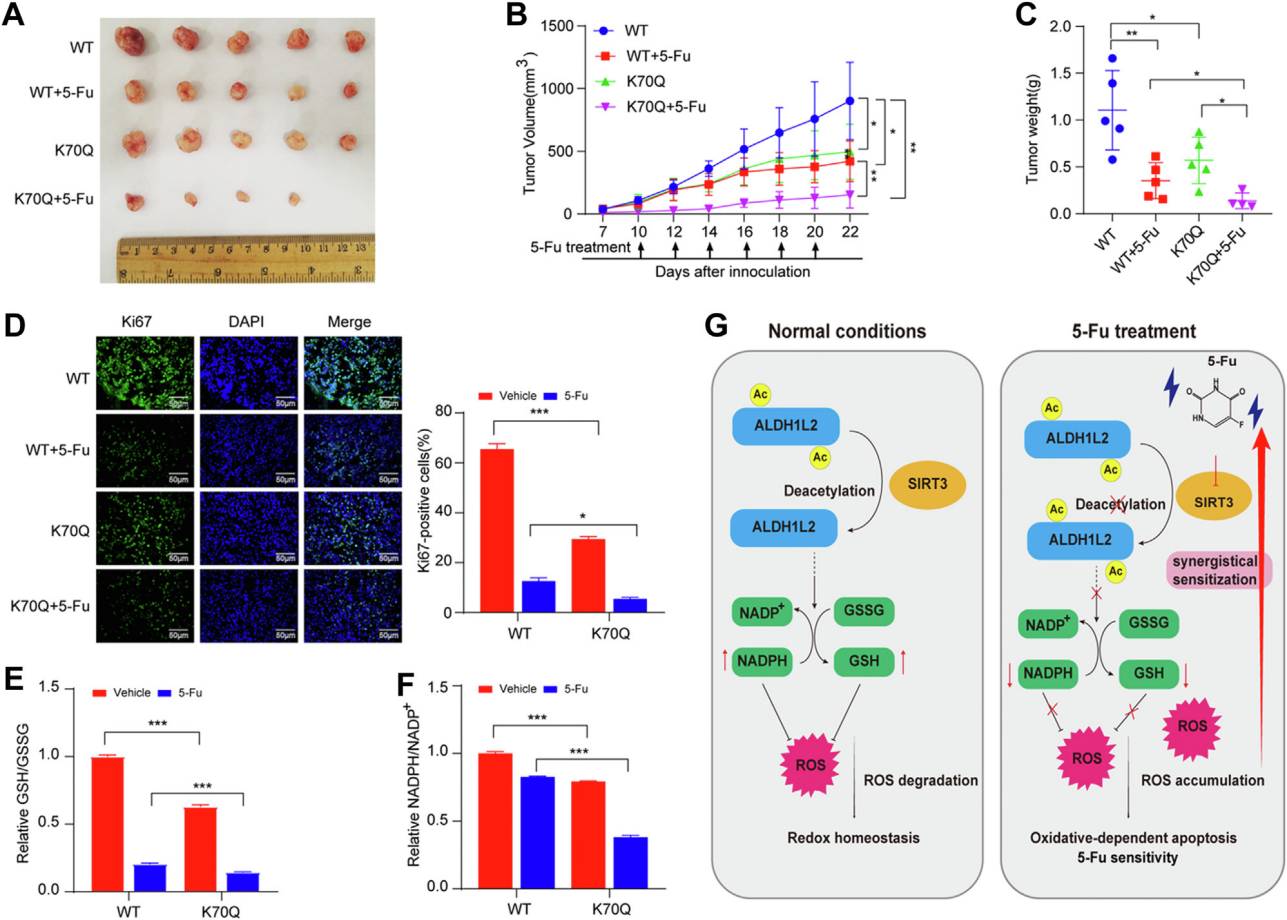
**ALDH1L2 K70Q mutant inhibits tumor growth and promotes 5-Fu sensitivity of CRC.***A*–*C*, tumor formation in nude mice (n =  5) injected with HCT116 cells reconstituted with ALDH1L2 WT or K70Q. Seven days after injection, mice were injected intraperitoneally with 50 mg/kg 5-Fu every 2 days as indicated. The tumors were dissected at day 22 after the injection. Pictures were taken at the sacrificed time point (*A*). Tumor volumes were measured at the indicated time points (*B*) and tumor mass was measured at the experimental endpoint (*C*). *D*, immunofluorescence staining analyses of xenograft samples from (*A*) using an anti-Ki67 antibody. Ki67-positive cells were quantified and presented in ten microscopic fields. *E* and *F*, relative GSH/GSSG (*E*) and NADPH/NADP^+^ (*F*) ratios in xenograft samples from (*A*) were determined. *G*, a working model of ALDH1L2 acetylation in regulating oxidative-induced apoptosis and 5-Fu sensitivity in CRC. Data are shown as mean ± S.D (n = 3). ∗*p* < 0.05; ∗∗*p* < 0.01; ∗∗∗*p* < 0.001. 5-FU, 5-fluorouracil; ALDH1L2, aldehyde dehydrogenase 1 family member L2; CRC, colorectal cancer.

**Figure S5:**
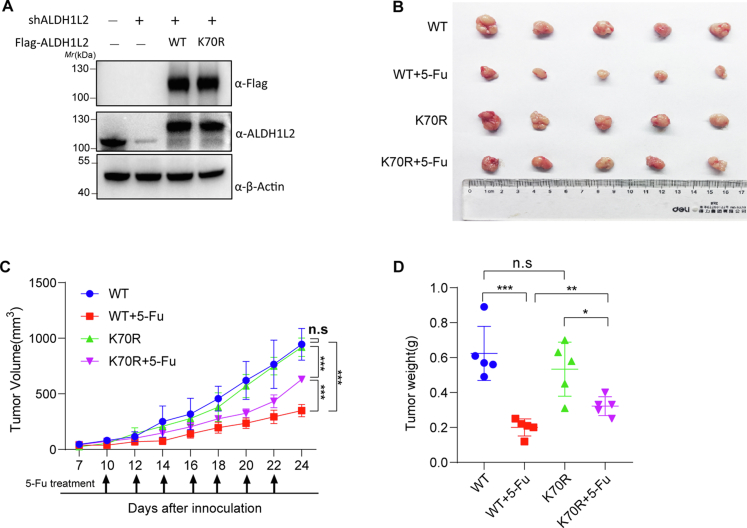
*A*, identification of reconstituted HCT116 cell lines. ALDH1L2-depleted HCT116 cells were reconstituted with empty vector, shRNA-resistant Flag-tagged WT and K70R mutant of ALDH1L2. Total cell lysates were prepared and detected by western blotting. *B*–*D*, Tumor formation in nude mice (n =  5) injected with HCT116 cells reconstituted with ALDH1L2 WT or K70R. Seven days after injection, mice were injected intraperitoneally with 50 mg/kg 5-Fu every two days as indicated. The tumors were dissected at day 24 after the injection. Pictures were taken at the sacrificed time point (*B*). Tumor volumes were measured at the indicated time points (*C*) and tumor mass was measured at the experimental endpoint (*D*). Data are shown as mean ± S.D (n = 3). ∗*p* < 0.05; ∗∗*p* < 0.01, ∗∗∗*p* < 0.001.

To further explore the role of K70 acetylation of ALDH1L2 on tumor growth and 5-Fu sensitivity *in vivo*, we injected reconstituted HCT116 cells with ALDH1L2 WT and ALDH1L2 K70Q into nude mice and analyzed their tumorigenesis and 5-Fu sensitivity. In contrast the WT cells, K70Q cells greatly repressed tumor growth, enhanced 5-Fu sensitivity, and disturbs cellular redox balance, as demonstrated by tumor volume and weight, Ki67 positivity, GSH/GSSG and NADPH/NADP^+^ ratios in tumor tissues (Fig. 6, *A*–*F*). In addition, considering that ALDH1L2 K70R mutant possesses equivalent activities to WT, but is no longer regulated by the 5-Fu-SIRT3 axis, we also found that HCT116 cells expressing ALDH1L2 K70R showed significantly less sensitivity to the 5-Fu than those expressing ALDH1L2 WT (Fig. S5, *A–D*). Collectively, these *in vivo* data confirm the aforementioned cellular data and support the notion that the K70Q mutant of ALDH1L2 inhibits tumor growth and improves the sensitivity of CRC to 5-Fu.

## Discussion

Recent studies have proposed that FOCM is a critical metabolic node in rapidly proliferating cancer cells, which helps support various biological processes, such as epigenetic modification, nucleotide synthesis, and antioxidant production (17). Many one carbon metabolic enzymes, including SHMT2, MTHFD2, and ALDH1L2, are highly expressed in human cancers, but how their activities are regulated is poorly understood (18). Lysine acetylation plays an extensive role in modulating metabolic enzyme activity. It is reported that acetylation of SHMT2 at K95 site inhibits its activity and promotes its degradation, thus suppressing the CRC progression (19). Also, SIRT4 senses folate availability and controls MTHFD2 protein stability through regulating K50 acetylation, leading to the suppression of breast cancer cell proliferation (20). At present, only few reports have investigated the role of ALDH1L2 in cancer progression and a recent study revealed that its abnormal expression was associated with radio-resistance in CRC (21). Bioinformatic analyses using public databases suggested that ALDH1L2 is highly expressed in CRC and is associated with poor prognosis (18, 22). In this study, we revealed that ALDH1L2 is overexpressed in CRC tissues and promotes CRC growth. Importantly, we uncovered, for the first time, the existence of acetylation modification of ALDH1L2, and identified K70 residue as its key acetylated site. Furthermore, we revealed that K70 acetylation inhibits ALDH1L2 activity and disturbs redox homeostasis, thus suppressing CRC cell proliferation and sensitizing CRC cells to 5-Fu both *in vitro* and *in vivo*. These data suggest a potential mechanism of metabolic adaption to extracellular stress in cancer cells.

Mitochondria produces a large amount of ROS under oxidative stress conditions, leading to mitochondrial dysfunction and eventually apoptosis (23, 24). SIRT3, mainly located in the mitochondria, appears to be a major regulator of oxidative stress. SIRT3 is an NAD+-dependent deacetylase which regulates many cellular processes, including cell metabolism, apoptosis, and cell survival, through deacetylating many mitochondrial proteins (25). Interestingly, SIRT3 may play a dual role in tumorigenesis and progression by acting as an oncogene or a tumor suppressor depending on its targets (26). However, how SIRT3 is involved in cancer is still not fully elucidated (27). We revealed that SIRT3 increases ALDH1L2 activity, thus enhances CRC proliferation, which is consistent with previous reports on the tumor-promoting role of SIRT3 in CRC (19, 28). In addition, K70Q mutant of ALDH1L2, with decreased ALDH1L2 enzymatic activity, increases ROS production and then promotes oxidative stress-induced apoptosis, highlighting the essential role of the SIRT3-ALDH1L2 axis in maintaining oxidative homeostasis in CRC.

Currently, the anti-metabolic drug 5-Fu is still widely used to treat various solid tumors, including CRC and gastric cancer (16). Although the combination of 5-Fu and other chemotherapeutic drugs can improve the prognosis of CRC patients, the frequent occurrence of 5-Fu resistance severely limits its clinical application (29). Therefore, understanding the mechanisms mediating 5-Fu resistance is a key step to predict or overcome this ticklish problem (29). We previously uncovered several novel mechanisms mediating 5-Fu resistance in CRC from epigenetic and tumor environment aspects (30, 31, 32, 33, 34, 35). Recently, metabolism reprogramming has emerged as a driving factor of chemoresistance, and the altered metabolic pathway helps cancer cells to proliferate faster than usual, adapt to nutrient-limited conditions, and form a chemoresistance phenotype (36, 37). Thus, recognizing metabolic alterations that sustaining drug resistance may help to develop effective methods for the prevention of drug resistance (36, 38, 39). Interestingly, it is reported that 5-Fu disrupts the metabolic flow of FOCM, implying that metabolic reprogramming may exist in this pathway and eventually lead to the resistance to 5-Fu (40, 41). However, to date, there are limited evidences to support the role of FOCM in 5-Fu resistance. In this article, we found that 5-Fu-induced oxidative stress and apoptosis can be rescued by SIRT3 overexpression. SIRT3 has been shown to react to multiple stimuli, including glucose starvation, hypoxia, and oxidative stress (42, 43, 44). We also proved that 5-Fu inhibits the expression of SIRT3 at both mRNA and protein levels, upregulating the acetylation level of ALDH1L2 and inhibiting its activity in CRC cells. Importantly, CRC cells expressing K70Q mutant of ALDH1L2 shows increased sensitive to 5-Fu treatment both *in vitro* and *in vivo*. Many literatures have reported that ROS can synergistically enhance the killing effect of chemotherapeutic agents, including 5-Fu, on tumor cells (45, 46). For example, Lin *et al* reported that acetylation at lysine 71 inactivates superoxide dismutase 1 and sensitizes tumor cells to genotoxic agents by increasing intracellular ROS levels (47). In this study, ALDH1L2 K70Q cells exhibits a higher ROS level than the WT group, which leads to a higher apoptosis rate and synergistically promotes 5-Fu sensitivity (Fig. 6*G*). However, HCT116 cells expressing ALDH1L2 K70R mutant, which possesses equivalent activities to WT and is no longer regulated by the 5-Fu-SIRT3 axis, exhibits comparable proliferative capacity and significantly less sensitivity to 5-Fu than those expressing ALDH1L2 WT. Additionally, it has been reported that PTMs of some enzymes, especially metabolic enzymes, affect the sensitivity of cancer cells to chemotherapeutic drugs (48, 49). Our data not only expand our understanding of the relationship between drug resistance and cancer metabolism, especially in the FOCM pathway, but also indicate that ALDH1L2 K70 could be a potential therapeutic site to prevent drug resistance.

This study focuses on the acetylation of ALDH1L2, an important one carbon metabolism enzyme in the mitochondria. Through an in-depth study of the regulatory role of SIRT3 on ALDH1L2, we revealed that K70 acetylation could inhibit ALDH1L2 activity, disturb redox homeostasis, and finally suppress CRC cell proliferation and sensitize CRC cells to 5-Fu. These results will help further to understand the role of ALDH1L2 in CRC progression. Notably, in addition to producing antioxidant NADPH, FOCM also contributes to nucleotide synthesis and methylation reactions. Whether or how acetylation of ALDH1L2 regulates thymidylate, purine synthesis, and methionine cycle requires further investigations. Additional efforts should be made to generate a site-specific antibody against K70 acetylated ALDH1L2, to determine the clinical relevance of ALDH1L2 K70 acetylation in 5-Fu-resistant tumors of CRC patients. In addition, our findings reveal a novel mechanism in the holistic picture of 5-Fu-induced tumor cell death, partially by inhibiting SIRT3-regulated one-carbon metabolism. Notably, SIRT3 expression was decreased by 5-Fu treatment in 5-Fu sensitive tumor cells, but not in 5-Fu-resistant cells, indicating that the SIRT3-ALDH1L2 axis is associated with the response of cancer cells to 5-Fu treatment.

In summary, our study not only provides a potential strategy for cancer therapy by targeting the K70 site of ALDH1L2 but also suggests that the combination of SIRT3 inhibitor and 5-Fu may overcome 5-Fu resistance.

## Experimental procedures

### Cell lines and clinical samples

The CRC cell lines (HCT116, CaCO2, RKO, SW480, SW620, and HCT8) were obtained from the American Type Culture Collection. Cells were cultured in Dulbecco's modified Eagle's medium supplemented with 10% fetal bovine serum and penicillin/streptomycin. All cell lines underwent authentication using short tandem repeat profiling and tested negative for *mycoplasma* contamination.

A total of 167 human CRC cases, including 143 paired tumor tissues and corresponding NCTs, were obtained from the Affiliated Hospital of Jiangnan University. The research was approved by both the local ethics committees and the International Agency for Research on Cancer Ethics Committee. All participants provided written, informed consent. The study was conducted following the Declaration of Helsinki and the International Ethical Guidelines for Biomedical Research Involving Human Subjects (*Council for International Organizations of Medical Sciences*).

### Western blotting and antibodies

Extracted proteins were separated by SDS-PAGE and transferred to a polyvinylidene fluoride membrane. The membrane was blocked with 5% nonfat milk and incubated with primary antibodies for β-actin (Proteintech, 81115-1-RR, 1:5000), ALDH1L2 (Proteintech, 21391-1-AP, 1:1000), acetylation (Cell Signaling Technology, 9441S, 1:1000), Flag (MBL, M185–3, 1:5000), SIRT3 (Santa cruz, sc-365175, 1:1000), HA (Proteintech, 51064-2-AP, 1:5000), His-Tag (Abmart, 2A8, 1:1000)

### qRT-PCR assay

Total RNA was extracted from cultured cells using TRIzol reagent (R401–01, Vazyme) and reverse transcribed into complementary DNA using the PrimeScript II 1st Strand Synthesis Kit (6210A, TaKaRa, Japan). qRT-PCR was performed on the ViiA7 real-time PCR system using the UltraSYBR Mixture (CW0957M, CWBIO) . The real-time PCR primers used are as follows: SIRT3 (forward): 5ʹ-CAGTCTGCCAAAGACCCTTC-3ʹ; SIRT3 (reverse): 5ʹ-AACACAA TGTCGGGCTTCAC-3ʹ; Actin (forward): 5ʹ-TCCATCATGAAGTGTGACG-3ʹ; Actin (reverse): 5ʹ-TACTCCTGCTTGCTGATCCAC-3ʹ.

### IHC staining

IHC assay was performed to detect the expression levels of ALDH1L2 protein in CRC tissues using tissue arrays constructed previously (50). In brief, paraffin-embedded tissues were sectioned at 4 μm. These slides were then incubated overnight at 4 °C with an anti-ALDH1L2 antibody. The following steps were carried out using the GTVision III Detection System/Mo&Rb (Gene Tech).

### Cell proliferation and colony formation assays

Cell proliferation assay was performed using the cell counting kit-8 (Dojindo) according to the manufacturer’s instructions. For colony formation assay, 800 to 1500 CRC cells were seeded in 6-well culture plates and cultured in media containing 10% fetal bovine serum for 10 days. The colonies were then fixed with formaldehyde and stained with 0.1% crystal violet to count the colony numbers.

### Co-immunoprecipitation

Cells were lysed in NP40 lysis buffer (50 mM Tris–HCl, pH 7.5, 150 mM NaCl, 0.1%-0.5% NP-40) containing protease inhibitor mixtures and 1 mM PMSF. Co-IP assays were carried out by incubating with anti-Flag M2 magnetic beads (A2220, Sigma-Aldrich) for over 4 h at 4 °C. After incubation, beads were washed three times with ice-cold NP40 buffer and the proteins were eluted in 1 × loading buffer by heating at 100 °C for 10 min. At last, Western blotting analyses were performed to analyze eluted proteins.

### Apoptosis analyses

For apoptosis analyses, CRC cells were treated with 5-Fu (0.8 μg/ml) for 48 h. These cells were then harvested by trypsinization, washed with ice-cold PBS, and fixed in 70% ice-cold ethanol in PBS. Apoptotic cells were detected using the Annexin V-FITC/PI Apoptosis Detection Kit (40302ES20, Yeasen).

### Reagents

5-Fu was purchased from Selleck (S1209). The complementary DNA encoding full-length human *ALDH1L2* and *SIRT3* were cloned into Flag- or HA-tagged vectors (pRK7-Flag and pcDNA-3HA). *ALDH1L2* point mutations were generated by Site-Directed Mutagenesis kit (C215–01, Vazyme). The siRNAs of *ALDH1L2* and *SIRT3* were purchased from RiboBio and the siRNA sequences are as follows: si*SIRT3*#1:5ʹ-GCTTGATGGACCAGACAAA-3ʹ; si*SIRT3*#2: 5ʹ-AAAGGTGGAAGAAGGTCCATATCTT-3ʹ; si*ALDH1L2*#1:5ʹ-CTGTGTTCAAG CTTCCTAAATGG-3ʹ; si*ALDH1L2*#2:5ʹ -CCCATGGATATAATTGAT AGTCC-3ʹ.

### Generation of reconstituted HCT116 cell lines

To deplete endogenous ALDH1L2, the shRNA targeting sequence (5ʹ-GCCATACC AGTGTTTCATAAA-3ʹ) was inserted into pMKO.1 retroviral vector (pMKO-shALDH1L2). pMKO-shALDH1L2 plasmids were cotransfected with pGAG and pVSVG into HEK293T cells. Retroviral supernatants were collected to infect HCT116 cells. Cells were selected with 2 μg/ml puromycin for 2 weeks. The knockdown efficiency was assessed by Western blotting. The rescued ALDH1L2 sequence (Flag-tagged WT or K70Q/K70R ALDH1L2) resistant to the ALDH1L2 shRNA was obtained *via* the mutation of the target sequence (5′-GCCgTAtCAaTGcTTtATcgA-3′). shRNA-resistant Flag-tagged WT or K70Q/K70R ALDH1L2 were cloned into the retroviral vector pQCXIH. Cells were infected with the retroviruses generated from these constructs and selected for two weeks with 250 μg/ml hygromycin.

### Mass spectrometry analyses

Total cell lysates of HEK293T cells transfected with Flag-ALDH1L2 were incubated with anti-Flag M2 magnetic beads for over 4 h at 4 °C. The bead–protein complexes were washed with ice-cold NP40 buffer three times. The complexes were then precipitated and eluted by Flag peptides (P9801, Beyotime). Finally, total eluted proteins were analyzed by mass spectrometry.

### Recombinant human ALDH1L2 expression and purification

DNA fragments of human *ALDH1L2* and its K70Q mutant were cloned into pET28a-sumo plasmids, and were expressed in *Escherichia coli* BL21 (DE3) using IPTG (Sigma-Aldrich, 1 mM) induction method. These bacteria were harvested and lysed. After centrifugation for 17,000 g for 1 h, the supernatants were collected and loaded onto a 1 ml Ni-Sepharose column His Trap-HP (GE HealthCare) by a peristaltic pump and gradient eluted by an ÄKTA FPLC system (GE HealthCare) with elution buffer (20 mM Tris–HCl pH 8.0, 500 mM NaCl, and 500 mM imidazole).

### ALDH1L2 activity detection

The dehydrogenase activities of ALDH1L2 were detected spectrophotometrically as described (6). Briefly, the reaction mixture contained 0.05 M Tris–HCl, pH 7.8, 100 mM 2-β-mercaptoethanol, 100 μM NADP^+^, 1 μg purified Flag-ALDH1L2 enzyme, and 10 μM 10-formyldideazafolate (10-FDDF, HY-143207, MedChemExpress) as a substrate. The monitored increase in absorbance at 340 nm (NADPH production) was used to calculate the dehydrogenase activity. All assays were carried out at 30 °C and the absorbance was measured by an Epoch 2 microplate reader (BioTek).

### NADPH/NADP^+^ and GSH/GSSG determination

HCT116 cells were seeded into 6-well plates, and cultured for 24 h in the presence and absence of 5-Fu for 48 h. Cells were harvested and NADPH/NADP^+^ and GSH/GSSG ratios were determined using an NADPH/NADP^+^ Assay Kit (S0179, Beyotime) and GSH/GSSG Assay Kit (S0053, Beyotime), respectively, according to the manufacturer’s instructions.

### Cellular ROS assay

Intracellular ROS levels were detected using the Reactive Oxygen Species Assay Kit (S0033, Beyotime) according to the manufacturer’s instructions. Briefly, HCT116 cells were seeded into 6-well plates, and cultured for 24 h in the presence or absence of 5-Fu for 72 h. Cells were then incubated with 10 μM dichlorodihydrofluorescein diacetate (DCFH-DA) (S0033S, Beyotime) at 37 °C for 20 min and subjected to flow cytometry analyses (BD).

### Identification of ALDH1L2 acetylation sites

HEK293T cells were transfected with Flag-tagged ALDH1L2 and cultured for 48 h. These transfected cells were then treated with 5 mM NAM (72345, Sigma-Aldrich) for 6 h before harvest. Total cells lysates were incubated with anti-Flag M2 magnetic beads for over 4 h and eluted by Flag peptides. Total eluted proteins were electrophoresed by SDS-PAGE gel and stained with Coomassie blue. The unique Flag-ALDH1L2 protein band was excised and digested with trypsin, followed by mass spectrometry analyses.

### Tumor formation assays in nude mouse models

HCT116 cells stably expressing ALDH1L2 (ALDH1L2 WT or ALDH1L2 K70Q/K70R) were subcutaneously injected into the same axillary subcutaneous of the same athymic male BALB/c nude mouse at 5 weeks of age (n= 5 for each group). Seven days after the injection of the HCT116 cells, mice were injected intraperitoneally with 50 mg/kg 5-Fu every 2days. Mice were sacrificed and examined for tumor growth 3 weeks after injection. All animal experiments were performed under the National Institutes of Health Guide for the Care and Use of Laboratory Animals and were approved by the Clinical Research Ethics Committees of Jiangnan University (JN.No20220515b0320730[151]).

### Statistical analyses

Data were presented as mean values ± SD. One-way ANOVA and two-tailed Student’s *t* test were performed to compare differences among different groups. The Kaplan–Meier method and log-rank test were used to compare survival between groups. Univariate and multivariate Cox proportional hazards regression models were applied to assess independent factors. All statistical analyses were performed using SPSS 20.0 software (IBM, SPSS, https://www.ibm.com/products/spss-statistics). A *p*-value below 0.05 (*p* < 0.05) was considered statistically significant.

## Data availability

All data pertinent to this work are contained within this manuscript or available upon request. For requests, please contact: Zhaohui Huang, zhaohuihuang@jiangnan.edu.cn.

## Supporting information

This article contains supporting information.
